# Race and Ethnicity, Socioeconomic Factors, and Epigenetic Age Acceleration in Survivors of Childhood Cancer

**DOI:** 10.1001/jamanetworkopen.2024.19771

**Published:** 2024-07-02

**Authors:** Cheng Chen, Noel-Marie Plonski, Qian Dong, Nan Song, Xijun Zhang, Hemang M. Parikh, Emily R. Finch, John Easton, Heather L. Mulder, Emily Walker, Geoffrey Neale, Yue Pan, Qian Li, Jinghui Zhang, Kevin Krull, Leslie L. Robison, Gregory T. Armstrong, Yutaka Yasui, Kirsten K. Ness, Melissa M. Hudson, Hui Wang, I-Chan Huang, Zhaoming Wang

**Affiliations:** 1 The Fourth Affiliated Hospital of Soochow University, SuZhou, Jiangsu, China; 2 State Key Laboratory of Systems Medicine for Cancer, Center for Single-Cell Omics, School of Public Health, Shanghai Jiao Tong University School of Medicine, Shanghai, China; 3 Department of Epidemiology and Cancer Control, St Jude Children’s Research Hospital, Memphis, Tennessee; 4 College of Pharmacy, Chungbuk National University, Cheongju, Korea; 5 Health Informatics Institute, Morsani College of Medicine, University of South Florida, Tampa; 6 Department of Computational Biology, St Jude Children’s Research Hospital, Memphis, Tennessee; 7 Hartwell Center, St Jude Children’s Research Hospital, Memphis, Tennessee; 8 Department of Biostatistics, St Jude Children’s Research Hospital, Memphis, Tennessee; 9 Department of Psychology, St Jude Children’s Research Hospital, Memphis, Tennessee; 10 Department of Oncology, St Jude Children’s Research Hospital, Memphis, Tennessee

## Abstract

**Importance:**

Current research in epigenetic age acceleration (EAA) is limited to non-Hispanic White individuals. It is imperative to improve inclusivity by considering racial and ethnic minorities in EAA research.

**Objective:**

To compare non-Hispanic Black with non-Hispanic White survivors of childhood cancer by examining the associations of EAA with cancer treatment exposures, potential racial and ethnic disparity in EAA, and mediating roles of social determinants of health (SDOH).

**Design, Setting, and Participants:**

In this cross-sectional study, participants were from the St Jude Lifetime Cohort, which was initiated in 2007 with ongoing follow-up. Eligible participants included non-Hispanic Black and non-Hispanic White survivors of childhood cancer treated at St Jude Children’s Research Hospital between 1962 and 2012 who had DNA methylation data. Data analysis was conducted from February 2023 to May 2024.

**Exposure:**

Three treatment exposures for childhood cancer (chest radiotherapy, alkylating agents, and epipodophyllotoxin).

**Main Outcomes and Measures:**

DNA methylation was generated from peripheral blood mononuclear cell-derived DNA. EAA was calculated as residuals from regressing Levine or Horvath epigenetic age on chronological age. SDOH included educational attainment, annual personal income, and the socioeconomic area deprivation index (ADI). General linear models evaluated cross-sectional associations of EAA with race and ethnicity (non-Hispanic Black and non-Hispanic White) and/or SDOH, adjusting for sex, body mass index, smoking, and cancer treatments. Adjusted least square means (ALSM) of EAA were calculated for group comparisons. Mediation analysis treated SDOH as mediators with average causal mediation effect (ACME) calculated for the association of EAA with race and ethnicity.

**Results:**

Among a total of 1706 survivors including 230 non-Hispanic Black survivors (median [IQR] age at diagnosis, 9.5 [4.3-14.3] years; 103 male [44.8%] and 127 female [55.2%]) and 1476 non-Hispanic White survivors (median [IQR] age at diagnosis, 9.3 [3.9-14.6] years; 766 male [51.9%] and 710 female [48.1%]), EAA was significantly greater among non-Hispanic Black survivors (ALSM = 1.41; 95% CI, 0.66 to 2.16) than non-Hispanic White survivors (ALSM = 0.47; 95% CI, 0.12 to 0.81). Among non-Hispanic Black survivors, EAA was significantly increased among those exposed to chest radiotherapy (ALSM = 2.82; 95% CI, 1.37 to 4.26) vs those unexposed (ALSM = 0.46; 95% CI, −0.60 to 1.51), among those exposed to alkylating agents (ALSM = 2.33; 95% CI, 1.21 to 3.45) vs those unexposed (ALSM = 0.95; 95% CI, −0.38 to 2.27), and among those exposed to epipodophyllotoxins (ALSM = 2.83; 95% CI, 1.27 to 4.40) vs those unexposed (ALSM = 0.44; 95% CI, −0.52 to 1.40). The association of EAA with epipodophyllotoxins differed by race and ethnicity (β for non-Hispanic Black survivors, 2.39 years; 95% CI, 0.74 to 4.04 years; β for non-Hispanic White survivors, 0.68; 95% CI, 0.05 to 1.31 years) and the difference was significant (1.77 years; 95% CI, 0.01 to 3.53 years; *P* for interaction = .049). Racial and ethnic disparities in EAA were mediated by educational attainment (<high school vs ≥college, ACME = 0.13; high school vs ≥college, ACME = 0.07; mediation = 22.71%) and ADI (ACME = 0.24; mediation = 22.16%).

**Conclusions and Relevance:**

In this cross-sectional study of childhood cancer survivors, race and ethnicity moderated the association of EAA with epipodophyllotoxin exposure and racial and ethnic differences in EAA were partially mediated by educational attainment and ADI, indicating differential treatment toxic effects by race and ethnicity. These findings suggest that improving social support systems may mitigate socioeconomic disadvantages associated with even greater accelerated aging and reduce health disparities among childhood cancer survivors.

## Key Points

**Question:**

Do the associations of epigenetic age acceleration (EAA) with cancer treatment exposures differ by race and ethnicity, and do social determinants of health (SDOH) account for the racial and ethnic disparity in EAA (if any)?

**Findings:**

In this cross-sectional study of 230 non-Hispanic Black and 1476 non-Hispanic White childhood cancer survivors, race and ethnicity moderated the associations of EAA with cancer treatment exposures, and racial and ethnic disparity in EAA was partially mediated by SDOH.

**Meaning:**

In this study, increased EAA associated with treatment differed between non-Hispanic Black and non-Hispanic White survivors; these findings suggest that improving social support systems may mitigate socioeconomic disadvantages associated with even greater accelerated aging among non-Hispanic Black survivors.

## Teaser

This cross-sectional study analyzes whether associations of epigenetic age acceleration (EAA) with cancer treatment vary by race and ethnicity among survivors of childhood cancer and whether socioeconomic factors mediate the associations.

## Introduction

Epigenetic age (EA) estimated with a DNA methylation (DNAm) profile is a well-established biomarker of aging.^1^ To date, at least a dozen epigenetic clocks have been proposed.^2,3,4,5^ The first generation of epigenetic clocks, represented by the Horvath clock,^3^ selected 5’-cytosine-phosphate-guanine-3’ to predict chronological age. The second generation, represented by the Levine clock (ie, DNAmPhenoAge),^4^ modeled physiological measurements and health outcomes. From these clocks, EA acceleration (EAA), such as EAA-Levine and EAA-Horvath, was estimated as the residual from a simple linear regression of EA on chronological age, representing the discrepancy between estimated EA and chronological age.

With improvements in survival rates for children who receive a diagnosis of cancer, the population of long-term survivors in the US has grown rapidly and is now estimated to exceed half a million.^6^ Adult survivors demonstrate a phenotype consistent with accelerated aging^7^ hallmarked by a substantial cumulative burden of chronic health conditions (CHCs), and such cumulative burden of CHCs shows racial and ethnic disparity.^8,9^ In a recent evaluation of EAA among non-Hispanic White participants in the St Jude Lifetime Cohort Study (SJLIFE),^10,11^ EAA-Levine was significantly greater in survivors than in controls without cancer and was associated with specific cancer treatment exposures, unfavorable health behaviors, and the presence of specific CHCs.^12^ Time-to-event analyses identified significant associations of EAA-Levine with the incidence of hypertension, myocardial infarction, obesity, obstructive pulmonary deficit, peripheral motor neuropathy, and peripheral sensory neuropathy, suggesting that EAA likely reflects the influence of genetics, treatment, and environmental exposures and may be predictive of CHCs. Another recent genetic study^13^ identified a novel locus indexed by rs732314 mapped to the *SELP* gene associated with EAA-Horvath among survivors. Taken together, these data support that EAA may be a promising biomarker to screen for the risk of adverse health outcomes, facilitating identification of individuals in need of behavioral,^14,15^ nutraceutical,^16,17^ senotherapeutic,^18,19^ or pharmaceutical^20,21,22,23^ interventions to prevent accelerated aging, and also potentially to serve as an intermediate measure indicative of intervention efficacies.

Published data^24^ have shown that in the general population (ie, those without cancer), low educational attainment or unhealthy lifestyles were independent predictors of greater EAA, suggesting that epigenetic clocks may elucidate the biological pathways underlying social and behavioral risk for premature aging. Furthermore, these data were supported by Yang et al^25^ who reported a significant association of cumulative pack-years of smoking with EAA.^4^ A more recent study^26^ demonstrated an association of high neighborhood deprivation and increased EAA. However, most literature describing these associations was limited to non-Hispanic White individuals. It is imperative to broaden the representation of races and ethnicities and increase inclusivity. Moreover, the association of socioeconomic adversity with EAA has not been explored among childhood cancer survivors.

To address these knowledge gaps, the present study aimed to evaluate EAA in non-Hispanic Black survivors, compare the EAA between non-Hispanic Black and non-Hispanic White survivors, investigate how associations of EAA with cancer treatment exposures differ by race and ethnicity, and evaluate the mediation effects of social determinants of health (SDOH) at either an individual or a community level on the racial and ethnic disparity in EAA.

## Methods

### Study Participants and Cancer Treatments

This cross-sectional study is part of the SJLIFE study, a retrospective cohort with prospective clinical follow-up and ongoing enrollment of survivors of childhood cancer treated at St Jude Children’s Research Hospital (SJCRH) between 1962 and 2012.^10,11^ Non-Hispanic Black and non-Hispanic White survivors with DNAm data were included in this analysis.^27^ Race and ethnicity was based on self-report. Cumulative doses of chemotherapy and region-specific radiation exposures were extracted from medical records.^28^ Three treatment modalities included chest radiotherapy, alkylating agents and epipodophyllotoxin, which were all previously associated with EAA.^12^ These analyses were limited to survivors at least 25 years of age when blood was drawn for EAA estimation because survivors younger than 25 years may have not yet completed their highest education (eg, college or postgraduate degree). The SJLIFE study protocol was approved by the institutional review board at SJCRH. All SJLIFE study participants provided written informed consent. This study followed the Strengthening the Reporting of Observational Studies in Epidemiology (STROBE) reporting guideline reporting guideline.

### DNA Methylation, EA, and EAA

Methylation profiling was generated using Infinium MethylationEPIC BeadChips version 1 (Illumina) on blood-derived DNA as previously described.^12,29^ EA was estimated using the Levine clock for the primary analysis because it is highly informative and slightly better than GrimAge^5^ in demonstrating the accelerated aging and estimating the risk of aging-related morbidity and premature mortality among childhood cancer survivors.^30^ In addition, the Horvath clock was analyzed as a comparison. EAA-Levine or EAA-Horvath was estimated as the residual from the fit of a simple linear regression of EA on age at blood draw.

### SDOH Factors

Three SDOH factors were ascertained at personal and neighborhood levels before or at the same time as the blood draw for methylation profiling.^27^ Personal level factors, obtained from self-report questionnaires, included educational attainment (<high school, high school, or ≥college) and annual personal income (none, >$1 to <$40 000, and ≥$40 000). Geocoded full residential addresses were used to obtain the area deprivation index (ADI) as a neighborhood socioeconomic status variable. ADI is a US census block level indicator based on 17 neighborhood-based socioeconomic status measures including income, employment, education, and housing characteristics that are collected as part of the American Community Survey.^31,32^ Census blocks are each assigned an ADI percentile ranking with minimum disadvantage in the first percentile and maximum disadvantage in the 100th percentile. To maximize statistical power, ADI was modeled as a continuous variable separately from personal level factors. However, to describe our cohort, 3 categories were also created (>75th percentile, 40th to 75th percentile, and <40th percentile) as high, moderate, and low deprivation, respectively.

### Body Mass Index and Smoking

Height by stadiometer and weight by electronic scale were measured to calculate body mass index (BMI; calculated as weight in kilograms divided by height in meters squared). BMI was dichotomized for analysis as less than 25.0 (underweight or normal weight) or 25.0 or greater (overweight or obese). Smoking status was assessed based on self-report questionnaires and categorized as never smoker, former smoker, or current smoker. BMI and smoking status were captured concurrently with the blood drawn for EAA estimation.

### Statistical Analysis

Pearson correlation coefficients (*r*) were used to measure the linear associations of EA with chronological age among non-Hispanic Black and non-Hispanic White survivors. To compare the annual change rate of EA between the 2 groups, a linear regression analysis using a general linear model (GLM) of EA against chronological age was conducted, and the difference in the annual change rate between racial and ethnic groups was evaluated by an interaction term (chronological age × race and ethnicity). To compare EAA between non-Hispanic Black and non-Hispanic White survivors, a regression analysis using a GLM of EAA for all survivors (denoted as the overall model) was employed, adjusting for sex, BMI, smoking and cancer treatment exposures, where race and ethnicity (non-Hispanic Black or non-Hispanic White) was the independent variable of interest, and adjusted least square means (ALSMs) of EAA was calculated for non-Hispanic Black and non-Hispanic White survivors. rs732314 was previously associated with EAA-Horvath but not EAA-Levine,^13^ so another model for EAA-Horvath additionally adjusted for rs732314 was performed for comparison. For more information on genotyping, please see the eMethods in Supplement 1. Associations of EAA with cancer treatment exposures were also evaluated in non-Hispanic Black and non-Hispanic White survivors separately. Specifically, to assess if the association of EAA with treatment differed by race and ethnicity, an interaction term (treatment exposure × race and ethnicity) was added to the overall GLM model. Linear regression with GLMs evaluated the association of EAA with SDOH factors, adjusting for race and ethnicity, sex, cancer treatment exposures, BMI, and smoking. Mediation analyses were performed with race and ethnicity as the independent variable with each SDOH factor as a mediator and EAA as the outcome. A 2-sided *P* < .05 was deemed as statistically significant. All analyses were performed with R statistical software version 3.6.1 (R Project for Statistical Computing).^33^ Data analysis was conducted from February 2023 to May 2024.

## Results

### Comparison of EA-Levine and EAA-Levine Between Non-Hispanic Black and Non-Hispanic White Survivors

A total of 1706 survivors, encompassing 230 non-Hispanic Black survivors (median [IQR] age at diagnosis, 9.5 [4.3-14.3] years; median [IQR] age at blood draw for DNAm, 34.6 [29.9-40.0] years; 103 male [44.8%] and 127 female [55.2%]) and 1476 non-Hispanic White survivors (median [IQR] age at diagnosis, 9.3 [3.9-14.6] years; median [IQR] age at blood draw, 36.6 [31.2-42.5] years; 766 male [51.9%] and 710 female [48.1%]) were included (Table 1 and eTable 1 in Supplement 1). SDOH factors differed between non-Hispanic Black and non-Hispanic White survivors, with non-Hispanic Black survivors reporting lower educational attainment, lower personal annual income, and residency in neighborhoods with more disadvantaged socioeconomic and physical environments as measured by the ADI.

**Table 1.** Characteristics of the Study Population

Characteristics	Participants, No. (%) (N = 1706)		*P* value^a^
	Non-Hispanic White (n = 1476)	Non-Hispanic Black (n = 230)	
Sex			
Male	766 (51.9)	103 (44.8)	.05^b^
Female	710 (48.1)	127 (55.2)	
Primary cancer diagnosis^c^			
Leukemia	493 (33.4)	44 (19.1)	<.001
Lymphoma	385 (26.1)	53 (23.0)	
Central nervous system tumors	118 (8.0)	21 (9.1)	
Other	480 (32.5)	112 (48.7)	
Chemotherapy and radiation therapy			
Chest radiotherapy	481 (32.6)	70 (30.4)	.56
Alkylating agent, classic	885 (60.0)	133 (57.8)	.58
Epipodophyllotoxins	422 (28.6)	57 (24.8)	.26
Social determinants of health factors			
Educational attainment			
<High school	129 (8.7)	36 (15.7)	<.001
High school	631 (42.8)	123 (53.5)	
≥College	650 (44.0)	58 (25.2)	
Unknown	66 (4.5)	13 (5.7)	
Personal income, $			
None	136 (9.2)	32 (13.9)	<.001
<40 000	865 (58.6)	151 (65.7)	
≥40 000	431 (29.2)	32 (13.9)	
Unknown	44 (3.0)	15 (6.5)	
Area deprivation index (national rank), median (IQR)	64 (43.0-82.0)	84 (64.8-95.0)	<.001
Age at diagnosis, median (IQR), y	9.3 (3.9-14.6)	9.5 (4.3-14.3)	.78
Age at survey, median (IQR), y	33.9 (29.5-40.3)	33.5 (28.7-37.8)	.06
Age at sampling, median (IQR), y	36.6 (31.2-42.5)	34.6 (29.9-40.0)	.02

^a^ For analysis, χ^2^test was used for categorical variables, Student *t* test for continuous variables, and Wilcoxon rank sum test for rank variables.

^b^ Compared with the subsamples of 4300 survivors without DNAm data (St Jude Lifetime Cohort Study data freeze 2020), the subsamples of 1706 survivors with DNAm data used in this analysis was not significantly different regarding sex.

^c^ The different proportions of childhood cancer diagnoses were because survivors of lymphoma were prioritized for initial cohort recruitment and the difference of race and ethnicity reflects the growth of the non-Hispanic Black population over time among St Jude Children’s Research Hospital patients (eTable 1 in Supplement 1).

There was a positive correlation of DNAmPhenoAge (ie, EA-Levine) with chronological age for non-Hispanic White survivors (*r* = 0.77) and non-Hispanic Black survivors (*r* = 0.74). The annual change rate of EA-Levine in a simple linear regression of EA-Levine on chronological age for non-Hispanic Black survivors (β = 0.85 years) was greater than for non-Hispanic White survivors (β = 0.77 years), but was not statistically significantly different (*P* for interaction = .10) (Figure, A). In a GLM adjusting for sex, chest radiotherapy, alkylating agents, epipodophyllotoxins, BMI, and smoking, EAA was significantly greater in non-Hispanic Black than non-Hispanic White survivors (β = 0.94 years; 95% CI, 0.18-1.70 years), and was greater in male vs female survivors (β = 1.08 years; 95% CI, 0.57-1.59 years), survivors with obesity vs without obesity (β = 0.60 years; 95% CI, 0.05-1.15 years), those exposed to chest radiotherapy vs those unexposed (β = 3.50 years; 95% CI, 2.95-4.05 years), those exposed to alkylating agents vs those unexposed (β = 1.69; 95% CI, 1.16-2.22 years), and those exposed to epipodophyllotoxins vs those unexposed (β = 0.87 years; 95% CI, 0.28-1.46 years) (Table 2 and eTable 2 in Supplement 1). On average, EAA was significantly greater in non-Hispanic Black survivors (ALSM  = 1.41; 95% CI, 0.66-2.16) than non-Hispanic White survivors (ALSM = 0.46, 95% CI, 0.12-0.81) (Figure, B).

**Figure.**
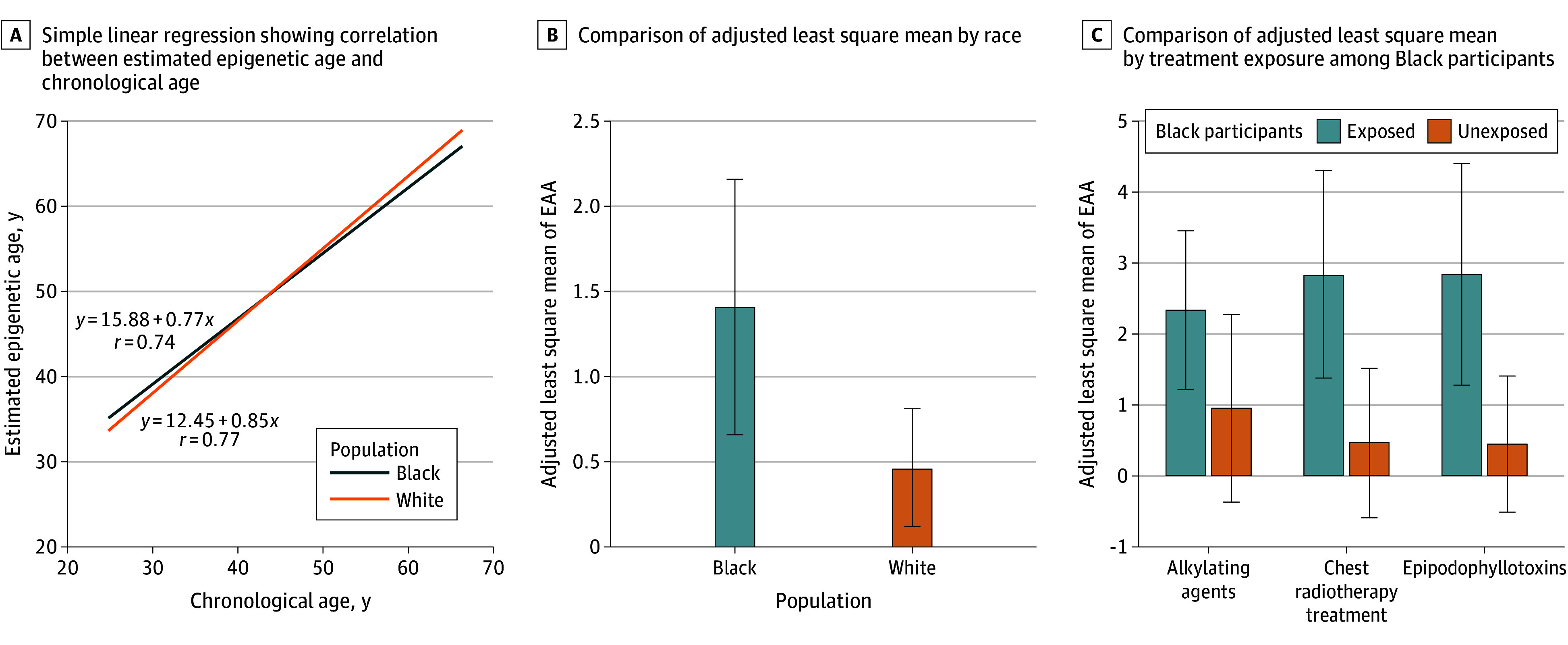
Epigenetic Age and Epigenetic Age Acceleration Among Non-Hispanic Black and Non-Hispanic White Survivors A, Simple linear regression showing the correlation between estimated epigenetic age based on the Levine clock (ie, DNAmPhenoAge) and chronological age among non-Hispanic Black and non-Hispanic White survivors. B, the adjusted least square mean (ALSM) of epigenetic age acceleration (EAA) based on the Levine clock among non-Hispanic Black and non-Hispanic White survivors. The ALSM was based on the overall model of EAA; race and ethnicity (non-Hispanic Black compared with non-Hispanic White) was the independent variable with covariates including sex (male vs female), cancer treatment exposures (chest radiotherapy, alkylating agents, and epipodophyllotoxins), body mass index, and smoking. C, The ALSM of EAA based on the Levine clock among non-Hispanic Black survivors between treatment-exposed vs treatment-unexposed groups. The ALSM was based on the stratified model (non-Hispanic Black survivors only) and the same set of covariates were considered.

**Table 2.** General Linear Models of Epigenetic Age Acceleration Among All Survivors, Non-Hispanic Black Survivors and Non-Hispanic White Survivors^a^

Coefficients	Epigentic age acceleration, β (95% CI), y		
	Overall (N = 1706)	Non-Hispanic White (n = 1476)	Non-Hispanic Black (n = 230)
Intercept	−3.42 (−4.09 to −2.75)	−3.54 (−4.25 to −2.83)	−2.78 (−4.60 to −0.96)
Non-Hispanic Black vs non-Hispanic White	0.94 (0.18 to 1.70)	NA	NA
Male vs female	1.08 (0.57 to 1.59)	1.11 (0.56 to 1.66)	1.34 (−0.09 to 2.77)
Chest radiotherapy	3.50 (2.95 to 4.05)	3.65 (3.06 to 4.24)	2.36 (0.81 to 3.91)
Alkylating agents	1.69 (1.16 to 2.22)	1.74 (1.17 to 2.31)	1.38 (−0.05 to 2.81)
Epipodophyllotoxins	0.87 (0.28 to 1.46)	0.68 (0.05 to 1.31)	2.39 (0.74 to 4.04)
Body mass index (≥25.0 vs <25.0)^b^	0.60 (0.05 to 1.15)	0.57 (−0.02 to 1.16)	0.84 (−0.69 to 2.37)
Smoking status (former or current vs never)	0.28 (−0.25 to 0.81)	0.22 (−0.34 to 0.78)	0.52 (−1.07 to 2.11)

Abbreviation: NA, not applicable.

^a^ In the overall model of epigenetic age acceleration, race and ethnicity (non-Hispanic Black compared with non-Hispanic White) was the independent variable with covariates including sex (male vs female), cancer treatment exposures (chest radiotherapy, alkylating agents, and epipodophyllotoxins), body mass index, and smoking. In the stratified models (non-Hispanic White only or non-Hispanic Black only), the same set of covariates were considered. The secondary analysis only including survivors of hematologic malignancies was also performed (eTable 2 in Supplement 1).

^b^ Body mass index was calculated as weight in kilograms divided by height in meters squared.

### Cancer Treatment Exposures and EAA-Levine Among Non-Hispanic Black and Non-Hispanic White Survivors

There were no significant differences in each of the 3 treatments between non-Hispanic Black and non-Hispanic White survivors (Table 1). Stratified analysis by racial and ethnic groups showed the same positive associations; treatment-exposed groups had greater EAA than nonexposed groups among non-Hispanic Black and non-Hispanic White survivors for each of the 3 treatments (Table 2). Among non-Hispanic Black survivors, EAA was greater in those exposed to chest radiotherapy (β = 2.36 years; 95% CI, 0.81 to 3.91 years) and epipodophyllotoxins (β = 2.39 years; 95% CI, 0.74 to 4.04 years). Among non-Hispanic White survivors, EAA was greater in those exposed to chest radiotherapy (β = 3.65 years; 95% CI, 3.06 to 4.24 years), alkylating agents (β = 1.74 years; 95% CI, 1.17 to 2.31 years), and epipodophyllotoxins (β = 0.68 years; 95% CI, 0.05 to 1.31 years). Moreover, the difference in the associations of EAA with epipodophyllotoxins for non-Hispanic Black and non-Hispanic White survivors was statistically significant (β =  1.77 years; 95% CI, 0.01 to 3.53 years; *P *for interaction = .049) (eTable 3 in Supplement 1) and the difference in the associations of chest-radiotherapy with EAA for non-Hispanic White and non-Hispanic Black survivors was not significant (β = −1.44 years; 95% CI, −3.09 to 0.21 years; *P* for interaction = .09) (eTable 4 in Supplement 1).

On average, among non-Hispanic Black survivors, EAA was greater in those exposed to chest radiotherapy (ALSM = 2.82; 95% CI, 1.37-4.26) vs those unexposed (ALSM = 0.46; 95% CI, −0.60 to 1.51), greater in those exposed to alkylating agents (ALSM, 2.33; 95% CI, 1.21 to 3.45) vs those unexposed (ALSM = 0.95; 95% CI, −0.38 to 2.27), and greater in those exposed to epipodophyllotoxins (ALSM = 2.83; 95% CI, 1.27 to 4.40) vs those unexposed (ALSM = 0.44; 95% CI, −0.52 to 1.40) (Figure, C). Among non-Hispanic White survivors, EAA was greater in those exposed to chest radiotherapy (ALSM = 2.27; 95% CI, 1.74 to 2.80) vs those unexposed (ALSM = −1.38; 95% CI, −1.76 to −1.00), greater in those exposed to alkylating agents (ALSM = 1.32; 95% CI, 0.92 to 1.71) vs those unexposed (ALSM = −0.43; 95% CI, −0.93 to 0.07), and greater in those exposed to epipodophyllotoxins (ALSM = 0.78; 95% CI, 0.21 to 1.35) vs those unexposed (ALSM = 0.11; 95% CI, −0.24 to 0.45) (eFigure 1 in Supplement 1).

### Racial and Ethnic Disparities Mediated by SDOH Factors in EAA-Levine

EAA was associated with educational attainment when comparing those who completed less than high school vs those who completed college or greater (β =  2.11 years; 95% CI, 1.03 to 3.19 years) and those who completed high school vs those who completed college or greater (β = 0.58 years; 95% CI, −0.11 to 1.27 years), which attenuated the association of EAA with race and ethnicity (from β = 0.94 years; 95% CI, 0.18 to 1.70 years to β = 0.80 years; 95% CI, −0.10 to 1.70 years) (Table 2 and Table 3). It is also notable that the association of EAA with BMI was substantially attenuated (from β = 0.60 years; 95% CI, 0.05-1.15 years to β = 0.32 years; 95% CI, −0.35 to 0.99 years). In contrast, the association of EAA with sex (male vs female) was slightly attenuated (from β = 1.08 years; 95% CI, 0.57 to 1.59 years to β = 0.90 years; 95% CI, 0.27 to 1.53 years). EAA was also associated with ADI per SD (β = 0.39 years; 95% CI, 0.06-0.72 years), and the association of EAA with racial and ethnic group was attenuated and became nonsignificant after adjusting for ADI (from β = 0.94 years; 95% CI, 0.18 to 1.70 years to β = 0.85 years; 95% CI, −0.05 to 1.75 years) (Table 2 and Table 3). Racial and ethnic disparities in EAA were mediated by educational attainment (<high school vs ≥college, ACME = 0.13; high school vs ≥college, ACME = 0.07; mediation = 22.71%) and ADI (ACME = 0.24; mediation = 22.16%) (Table 4).

**Table 3.** Epigenetic Age Acceleration, Educational Attainment, and Area Deprivation Index Among All Survivors

Coefficients	Epigenetic age acceleration, β (95% CI)
Educational attainment	
Intercept	−3.42 (−4.26 to −2.58)
Educational attainment (<high school vs ≥ college)	2.11 (1.03 to 3.19)
Educational attainment (high school vs ≥ college)	0.58 (−0.11 to 1.27)
Non-Hispanic Black vs non-Hispanic White	0.80 (−0.10 to 1.70)
Male vs female	0.90 (0.27 to 1.53)
Chest radiotherapy	2.96 (2.29 to 3.63)
Alkylating agents	1.85 (1.20 to 2.50)
Epipodophyllotoxins	0.49 (−0.22 to 1.20)
Body mass index (≥25.0 vs <25.0)^a^	0.32 (−0.35 to 0.99)
Smoking status (former or current vs never)	0.26 (−0.41 to 0.93)
Area Deprivation Index	
Intercept	−3.15 (−3.97 to −2.33)
Area Deprivation Index	0.39 (0.06 to 0.72)
Non-Hispanic Black vs non-Hispanic White	0.85 (−0.05 to 1.75)
Male vs female	0.92 (0.29 to 1.55)
Chest radiotherapy	3.08 (2.41 to 3.75)
Alkylating agents	1.80 (1.17 to 2.43)
Epipodophyllotoxins	0.54 (−0.15 to 1.23)
Body mass index (≥25.0 vs <25.0)^a^	0.39 (−0.28 to 1.06)
Smoking status (former or current vs never)	0.49 (−0.16 to 1.14)

^a^ Body mass index was calculated as weight in kilograms divided by height in meters squared.

**Table 4.** Mediation of Epigenetic Age Acceleration in Racial and Ethnic Disparities by Educational Attainment and Area Deprivation Index^a^

Mediator	Average causal mediation effect	Average direct effect	Total effect^b^	Mediation, %^c^	*P* value
Educational attainment (<high school vs ≥ college)	0.13	0.74	0.88	14.50	.05
Educational attainment (high school vs ≥ college)	0.07	0.74	0.81	8.88	.12
Educational attainment (total)	0.22	0.74	0.96	22.71	NA
Area Deprivation Index (continuous variable)	0.24	0.85	1.09	22.16	.03

Abbreviation: NA, not applicable.

^a^ Mediation R package was used for the mediation analysis including 2 regression models: (1) a generalized linear regression model with epigenetic age acceleration as an outcome variable, each social determinant of health factor as a mediator variable, and adjusted for covariates (age, sex, cancer treatment exposures, body mass index, and smoking) and (2) a generalized linear regression model with each social determinant of health factor as an outcome variable and race and ethnicity as an independent variable.

^b^ Calculated by summing average causal mediation effect and average direct effect.

^c^ Calculated by average causal mediation effect divided by total effect (the sum of average causal mediation and average direct effect).

### Comparative Analyses of EAA-Horvath

There was a positive correlation of EA-Horvath with chronological age for non-Hispanic White survivors (*r* = 0.84) and non-Hispanic Black survivors (*r* = 0.78). The age slope of EA-Horvath for non-Hispanic White survivors (β = 0.59) was not different from non-Hispanic Black survivors (β = 0.54) (*P* for interaction = .11) (eFigure 2 in Supplement 1). In a GLM adjusting for sex, chest radiotherapy, alkylating agents, epipodophyllotoxins, BMI, and smoking, EAA-Horvath was greater in non-Hispanic Black than non-Hispanic White survivors (β = 0.44 years; 95% CI, 0.01 to 0.87 years), male vs female survivors (0.36 years; 95% CI, 0.07 to 0.65 years), those exposed to chest radiotherapy vs those unexposed (β = 1.19 years; 95% CI, 0.88 to 1.50 years), and in those exposed to alkylating agents (β = 0.44 years; 95% CI, 0.15 to 0.73 years), but was lower in smokers vs nonsmokers (β = −0.47 years; 95% CI, −0.18 to −0.76 years) (eTable 5 in Supplement 1). On average, EAA-Horvath was significantly greater in non-Hispanic Black survivors (ALSM = 0.52; 95% CI, 0.10 to 0.95) than non-Hispanic White survivors (ALSM = 0.09; 95% CI, −0.11 to 0.28) (eFigure 3 in Supplement 1).

Among non-Hispanic Black survivors, EAA-Horvath was not associated with chest radiotherapy, alkylating agents, or epipodophyllotoxins. Among non-Hispanic White survivors, EAA-Horvath was greater in those exposed to chest radiotherapy (β = 1.21 years; 95% CI, 0.88-1.54 years) and those exposed to alkylating agents (β = 0.40 years; 95% CI, 0.09-0.71 years); however, no difference was observed among those exposed vs not exposed to epipodophyllotoxins (eTable 6 in Supplement 1).

There were no associations of SDOH factors with EAA-Horvath. However, EAA-Horvath was associated with rs732314 per copy of rs732314-C allele (β = 0.42 years; 95% CI, 0.22-0.62 years) in the overall study population including both non-Hispanic Black and non-Hispanic White survivors (eTable 7 in Supplement 1). EAA-Horvath was greater in non-Hispanic Black survivors than non-Hispanic White survivors before adjusting for rs732314 (β = 0.44 years; 95% CI, 0.01-0.87 years) (eTable 5 in Supplement 1) and became nonsignificant with adjustment for rs732314 (β = 0.35 years; 95% CI, −0.08 to 0.78 years) (eTable 7 in Supplement 1). The allele frequency for rs732314-C allele was greater in non-Hispanic Black survivors than non-Hispanic White survivors (0.62 vs 0.51; *P* < .001).

## Discussion

To our knowledge, this is the first cross-sectional study evaluating EAA in non-Hispanic Black survivors of childhood cancer. We observed racial and ethnic disparities in both EAA-Levine and EAA-Horvath; specifically, there was greater EAA among non-Hispanic Black survivors compared with non-Hispanic White survivors. The racial and ethnic disparity in EAA-Levine was partially mediated by SDOH factors, specifically, educational attainment at the personal level and ADI at neighborhood and geographic level. In contrast, the racial and ethnic disparity in EAA-Horvath was partially accounted for by rs732314.

We noted that the correlation between EA and chronological age for both the Levine and Horvath clocks was similar between non-Hispanic Black survivors and non-Hispanic White survivors, suggesting that epigenetic clocks are valid and robust across survivors of different races and ethnicities, which was consistent with previous reports in the general population.^34^ The association of cancer treatment exposures with EAA-Levine was moderated by race and ethnicity (ie, it varied between non-Hispanic Black survivors and non-Hispanic White survivors). Specifically, chest radiotherapy exposure was associated with a moderately larger EAA difference among non-Hispanic White survivors, whereas epipodophyllotoxins exposure was associated with a much larger EAA difference among non-Hispanic Black survivors. In contrast, the association of treatment exposures with EAA-Horvath was similar between non-Hispanic Black and non-Hispanic White survivors. SDOH factors such as educational attainment and ADI mediated the racial and ethnic disparity in EAA-Levine, suggesting that targeted interventions providing personal and neighborhood-specific resources (eg, coping skills and guidance to survivors with lower educational attainment and those living in neighborhoods with more deprivation) may mitigate accelerated aging among survivors. Our study revealed that educational attainment, rather than personal income, was significantly associated with EAA. It is also critical to address structural inequities as one of the root causes of health disparities, typically through policy amendments to improve employment and education opportunities, housing conditions, transportation, and accessibility and quality of health care for survivors who live in rural and remote geographical areas or deprived neighborhoods.

Previous studies^24^ demonstrated an association of educational attainment with EAA in the general population. Our findings extended the findings from a recent study^26^ that reported an association of a greater ADI with various methylation-based markers of aging, including EAA-Levine but not EAA-Horvath in the general population, and provided additional insights into survivors. The specific associations using the Levine clock but not the Horvath clock is plausible considering that the Levine clock models the aging-related composite physiological estimates rather than solely the chronological age. Intuitively, one might expect the Levine clock to be more sensitive to the biological outcomes associated with social adversity such as lower educational attainment or living in a socioeconomically disadvantaged neighborhood. In the field of medicine,^35^ race and ethnicity has been affirmatively defined as a social construct but it correlates with genetic ancestry. Here, we showed that racial and ethnic disparity in EAA-Horvath was partially explained by the difference in risk allele frequency of the genetic variant (rs732314).^13^

### Limitations

Our study had several limitations. First, low statistical power due to the sample size of non-Hispanic Black survivors precluded evaluation of the impact of additional cancer treatments; therefore, we followed the strategy of evaluating and comparing the effect of treatment exposures previously established among non-Hispanic White survivors. The association of chest radiotherapy with EAA showed a suggestive difference for increased accelerated aging in non-Hispanic White compared with non-Hispanic Black survivors, which is interesting given the differences in skin pigmentation that might modify cellular effects of radiotherapy.^36,37^ However, this should be further investigated in larger populations. The small sample size of non-Hispanic Black survivors also precluded evaluation of the mediation of EAA on the association of race and ethnicity and CHCs. Future studies are needed to investigate the associations of EAA with CHCs among socially disadvantaged non-Hispanic Black survivors. Second, the measurement of EAA was limited to a single time point due to the cross-sectional study design. Ideally, a longitudinal study design with multiple repeated measurements of EAA are needed to assess the change and trajectory of EAA based on a more comprehensive set of SDOH factors such as occupation, access to medical services, health insurance, and rurality. Third, we caution that statistical tests on the associations of EAA with 3 treatment exposures or 3 SDOH factors as well as 2 mediation analyses did not adjust for multiple comparisons. Fourth, other racial and ethnic groups should be considered in future studies. Fifth, our findings were based on a single-center cohort that may not be representative of the entire population of childhood cancer survivors. Validation of our findings in multicenter cohorts, (eg, Childhood Cancer Survivor Study^38^) is warranted.

## Conclusions

In summary, our findings provide substantial new knowledge regarding EAA among survivors of childhood cancer. Observed racial and ethnic disparity in EAA-Levine was partially mediated by SDOH including educational attainment and ADI. This finding suggests that changes in the social support system at both the personal and community levels may mitigate socioeconomic disadvantages that may contribute to the development of an aging phenotype. Longitudinal intervention trials are needed to test this strategy in the future.

## Notes

Supplement 1.
eMethods.

eReferences.
**eTable 1.** Characteristics of SJLIFE Survivors With and Without DNA Methylation Data **eTable 2.** General Linear Models of EAA Among All, NHB and NHW Survivors With Childhood Hematological Malignancies **eTable 3.** Interaction Between Racial/Ethnic Groups and Epipodophyllotoxins for Association With Levine-EAA **eTable 4.** Interaction Between Racial/Ethnic Groups and Chest-RT for Association With Levine-EAA **eFigure 1.** Adjusted Least Square Mean (ALSM) of Epigenetic Age Acceleration (EAA) Based on Levine’s Clock Among Non-Hispanic White (NHW) Survivors Between Treatment-Exposed Versus Treatment-Unexposed Groups **eFigure 2.** Linear Correlation Between Epigenetic Age Based on Horvath’s Clock and Chronological Age Among NHB and NHW Survivors Respectively **eTable 5.** General Linear Model of EAA-Horvath Among All Survivors **eFigure 3.** Adjusted Least Square Mean (ALSM) of Epigenetic Age Acceleration (EAA) Based on Horvath’s Clock Among NHB and NHW Survivors **eTable 6.** Stratified Analysis of EAA-Horvath by Racial/Ethnic Groups for Treatment Exposures **eTable 7.** rs732314 Is Associated With EAA-Horvath Among All Survivors

Supplement 2.
Data Sharing Statement
