# Supplementary material for: Race and Ethnicity, Socioeconomic Factors, and Epigenetic Age Acceleration in Survivors of Childhood Cancer
Source: JAMA Netw Open. 2024 Jul 2;7(7):e2419771. doi: 10.1001/jamanetworkopen.2024.19771 (PMC11220564; doi:10.1001/jamanetworkopen.2024.19771)
Supplement: Supplement 1. — eMethods. eReferences. eTable 1. Characteristics of SJLIFE Survivors With and Without DNA Methylation Data eTable 2. General Linear Models of EAA Among All, NHB and NHW Survivors With Childhood Hematological Malignancies eTable 3. Interaction Between Racial/Ethnic Groups and Epipodophyllotoxins for Association With Levine-EAA eTable 4. Interaction Between Racial/Ethnic Groups and Chest-RT for Association With Levine-EAA eFigure 1. Adjusted Least Square Mean (ALSM) of Epigenetic Age Acceleration (EAA) Based on Levine’s Clock Among Non-Hispanic White (NHW) Survivors Between Treatment-Exposed Versus Treatment-Unexposed Groups eFigure 2. Linear Correlation Between Epigenetic Age Based on Horvath’s Clock and Chronological Age Among NHB and NHW Survivors Respectively eTable 5. General Linear Model of EAA-Horvath Among All Survivors eFigure 3. Adjusted Least Square Mean (ALSM) of Epigenetic Age Acceleration (EAA) Based on Horvath’s Clock Among NHB and NHW Survivors eTable 6. Stratified Analysis of EAA-Horvath by Racial/Ethnic Groups for Treatment Exposures eTable 7. rs732314 Is Associated With EAA-Horvath Among All Survivors [file jamanetwopen-e2419771-s001.pdf]

Supplemental Online Content

Chen C, Plonski N-M, Dong Q, et al. Race and ethnicity, socioeconomic factors, and epigenetic age acceleration in survivors of childhood cancer. *JAMA Netw. Open*. 2024;7(6):e2418831. doi:10.1001/jamanetworkopen.2024.19771

eMethods

eReferences

eTable 1. Characteristics of SJLIFE Survivors With and Without DNA Methylation Data

eTable 2. General Linear Models of EAA Among All, NHB and NHW Survivors With Childhood Hematological Malignancies

eTable 3. Interaction Between Racial/Ethnic Groups and Epipodophyllotoxins for Association With Levine-EAA

eTable 4. Interaction Between Racial/Ethnic Groups and Chest-RT for Association With Levine-EAA

eFigure 1. Adjusted Least Square Mean (ALSM) of Epigenetic Age Acceleration (EAA) Based on Levine's Clock Among Non-Hispanic White (NHW) Survivors Between Treatment-Exposed Versus Treatment-Unexposed Groups

eFigure 2. Linear Correlation Between Epigenetic Age Based on Horvath's Clock and Chronological Age Among NHB and NHW Survivors Respectively

eTable 5. General Linear Model of EAA_Horvath Among All Survivors

eFigure 3. Adjusted Least Square Mean (ALSM) of Epigenetic Age Acceleration (EAA) Based on Horvath's Clock Among NHB and NHW Survivors

eTable 6. Stratified Analysis of EAA-Horvath by Racial/Ethnic Groups for Treatment Exposures

eTable 7. rs732314 Is Associated With EAA-Horvath Among All Survivors

This supplemental material has been provided by the authors to give readers additional information about their work.

eMethods

Genotyping was based on whole-genome sequencing data from blood-derived DNA for SJLIFE survivors as previously described[1,2]. Genotypes were extracted for rs732314 (chr1:169630016:C:T), previously associated with EAA-Horvath, and were subsequently coded as 0, 1 and 2 for the following genotypes: TT, CT, and CC, respectively. Note that rs732314-C is the risk increasing allele[3]. Genotypes for rs732314 partially accounted for the association between race/ethnicity and EAA-Horvath.

eReferences

1. Qin N, Wang Z, Liu Q, et al. Pathogenic Germline Mutations in DNA Repair Genes in Combination With Cancer Treatment Exposures and Risk of Subsequent Neoplasms Among Long-Term Survivors of Childhood Cancer. *J Clin Oncol* 2020; **38**(24): 2728-40.
2. Wang Z, Wilson CL, Easton J, et al. Genetic Risk for Subsequent Neoplasms Among Long-Term Survivors of Childhood Cancer. *J Clin Oncol* 2018; **36**(20): 2078-87.
3. Dong Q, Song N, Qin N, et al. Genome-wide association studies identify novel genetic loci for epigenetic age acceleration among survivors of childhood cancer. *Genome Med* 2022; **14**(1): 32.

© 2024 Chen C et al. *JAMA Network Open*

eTable 1. Characteristics of SJLIFE Survivors With and Without DNA Methylation Data

Characteristics	Childhood cancer survivors without DNAm No. (%)	Childhood cancer survivors with DNAm No. (%)	P*
Total	4300 (71.6)	1706 (28.4)	
Sex			0.24
Female	2038 (47.4)	837 (49.1)	
Male	2262 (52.6)	869 (50.9)	
Race/Ethnicity			<0.0001
White, Non Hispanic	3156 (73.4)	1476 (86.5)	
Black, Non Hispanic	752 (17.5)	230 (13.5)	
Others	392 (9.1)	0 (0.0)	
Diagnosis			<0.0001
Leukemia	1442 (33.5)	537 (31.5)	
Lymphoma	509 (11.8)	438 (25.7)	
Sarcoma	457 (10.6)	249 (14.6)	
CNS tumor	817 (19.0)	139 (8.1)	
Embryonal	515 (12.0)	231 (13.5)	
Others	560 (13.0)	112 (6.6)	
Median age at diagnosis, IQR, years	5.5 (2.4-11.6)	9.4 (3.9, 14.5)	<0.0001

*Chi-square test for categorical variables, Student's t-test for continuous variables, and Wilcoxon rank sum test for rank variables;
Abbreviations: NHW, non-Hispanic White; NHB, non-Hispanic Black; SDOH, social determinants of health; ADI, area deprivation index

© 2024 Chen C et al. *JAMA Network Open*

eTable 2. General Linear Models of EAA Among All, NHB and NHW Survivors With Childhood Hematological Malignancies

	Overall (n=975)		NHW (n=878)		NHB (n=97)	
**Coefficients**	**Estimate**	**95% CI**	**Estimate**	**95% CI**	**Estimate**	**95% CI**
Intercept	-3.58	-4.54, -2.62	-3.37	-4.37, -2.37	-3.54	-6.56, -0.52
NHB vs. NHW	1.96	0.86, 3.06	NA	NA	NA	NA
Male vs. female	1.33	0.68, 1.98	1.32	0.63, 2.01	1.81	-0.23, 3.85
Chest radiotherapy	3.53	2.82, 4.24	3.63	2.89, 4.37	2.65	0.36, 4.94
Alkylating agents	1.23	0.54, 1.92	1.28	0.55, 2.01	0.87	-1.38, 3.12
Epipodophyllotoxins	0.39	-0.32, 1.10	0.39	-0.35, 1.13	0.91	-1.27, 3.09
BMI (>=25.0 vs. <25.0)	0.27	-0.44, 0.98	0.24	-0.50, 0.98	0.33	-1.87, 2.53
Smoking status (former/current vs. never)	0.41	-0.26, 1.08	0.34	-0.37, 1.05	0.77	-1.50, 3.04

Abbreviations: EAA, epigenetic age acceleration; NHW, non-Hispanic White; NHB, non-Hispanic Black.

eTable 3. Interaction Between Racial/Ethnic Groups and Epipodophyllotoxins for Association With Levine-EAA

**Coefficients**	**Estimate**	**95% CI**	**P**
Intercept	-3.49	-4.16, -2.82	**<0.001**
Male vs. female	1.10	0.59, 1.61	**<0.001**
NHB vs. NHW	0.51	-0.37, 1.39	0.25
Epipodophyllotoxins	0.66	0.03, 1.29	**0.04**
Alkylating agents	1.69	1.16, 2.22	**<0.001**
Chest radiotherapy	3.49	2.94, 4.04	**<0.001**
BMI (>=25.0 vs. <25.0)	0.60	0.05, 1.15	**0.03**
Smoking status (former/current vs. never)	0.26	-0.27, 0.79	0.33
Racial/ethnic groups_Epipodophyllotoxins	1.77	0.01, 3.53	**0.05**

Abbreviations: NHB, non-Hispanic Black; NHW, non-Hispanic White.

© 2024 Chen C et al. *JAMA Network Open*

eTable 4. Interaction Between Racial/Ethnic Groups and Chest-RT for Association With Levine-EAA

Coefficients	Estimate	95% CI	P
**Intercept**	-3.62	-4.31, -2.93	**<0.001**
**Male vs. female**	1.12	0.61, 1.63	**<0.001**
**NHB vs. NHW**	1.39	0.47, 2.31	**0.003**
**Chest radiotherapy**	3.68	3.09, 4.27	**<0.001**
**Epipodophyllotoxins**	0.89	0.30, 1.48	**0.003**
**Alkylating agents**	1.69	1.16, 2.22	**<0.001**
**BMI (>=25.0 vs. <25.0)**	0.59	0.04, 1.14	**0.03**
**Smoking status (former/current vs. never)**	0.27	-0.26, 0.80	0.31
**Racial/ethnic groups_Chest radiotherapy**	-1.44	-3.09, 0.21	0.09

Abbreviations: EAA, epigenetic age acceleration; NHB, non-Hispanic Black; NHW, non-Hispanic White.

© 2024 Chen C et al. *JAMA Network Open*

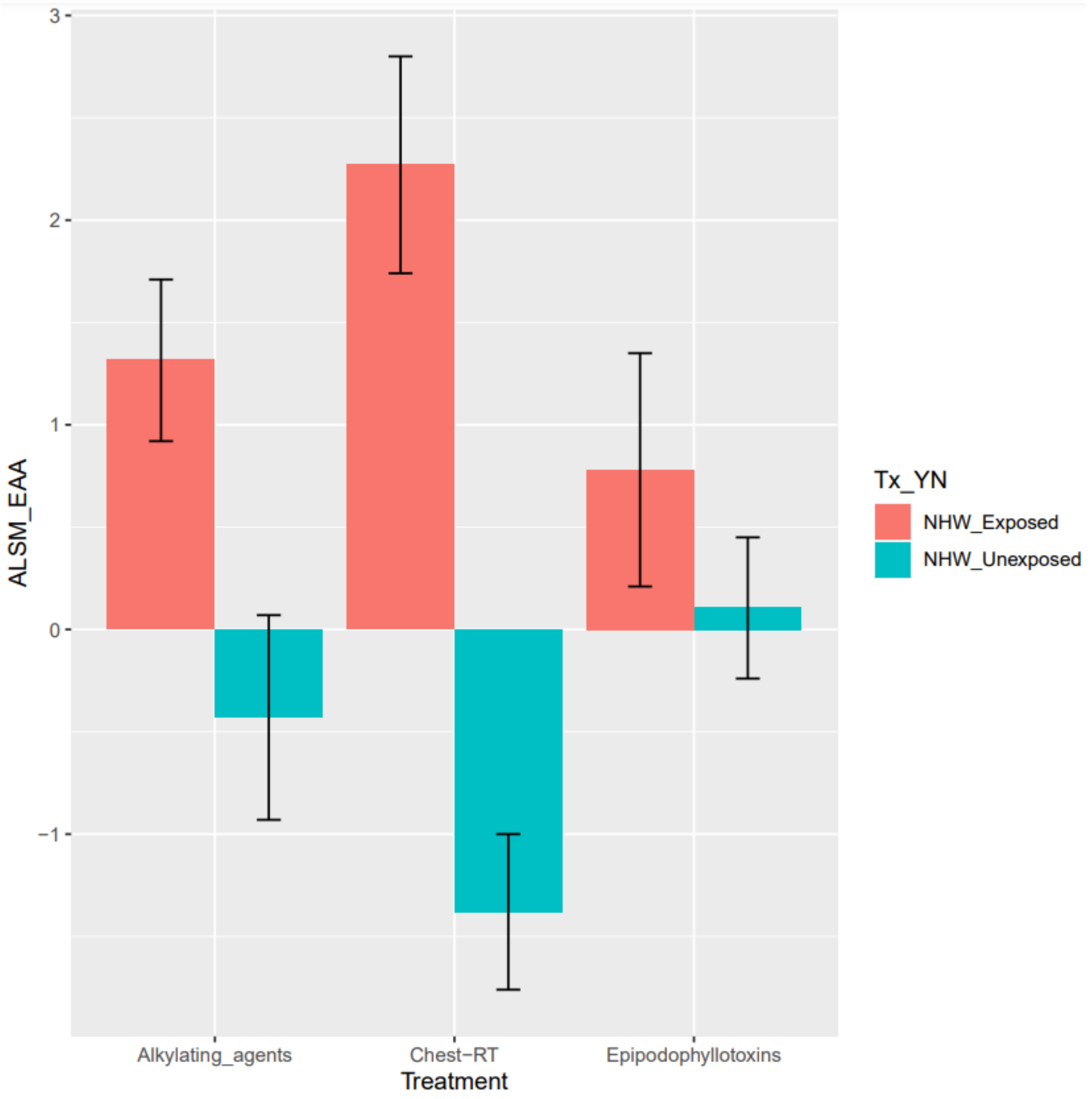

eFigure 1. Adjusted Least Square Mean (ALSM) of Epigenetic Age Acceleration (EAA) Based on Levine's Clock Among Non-Hispanic White (NHW) Survivors Between Treatment-Exposed Versus Treatment-Unexposed Groups

Three cancer treatment exposures were included: alkylating agents, chest-RT and epipodophyllotoxins.

© 2024 Chen C et al. *JAMA Network Open*

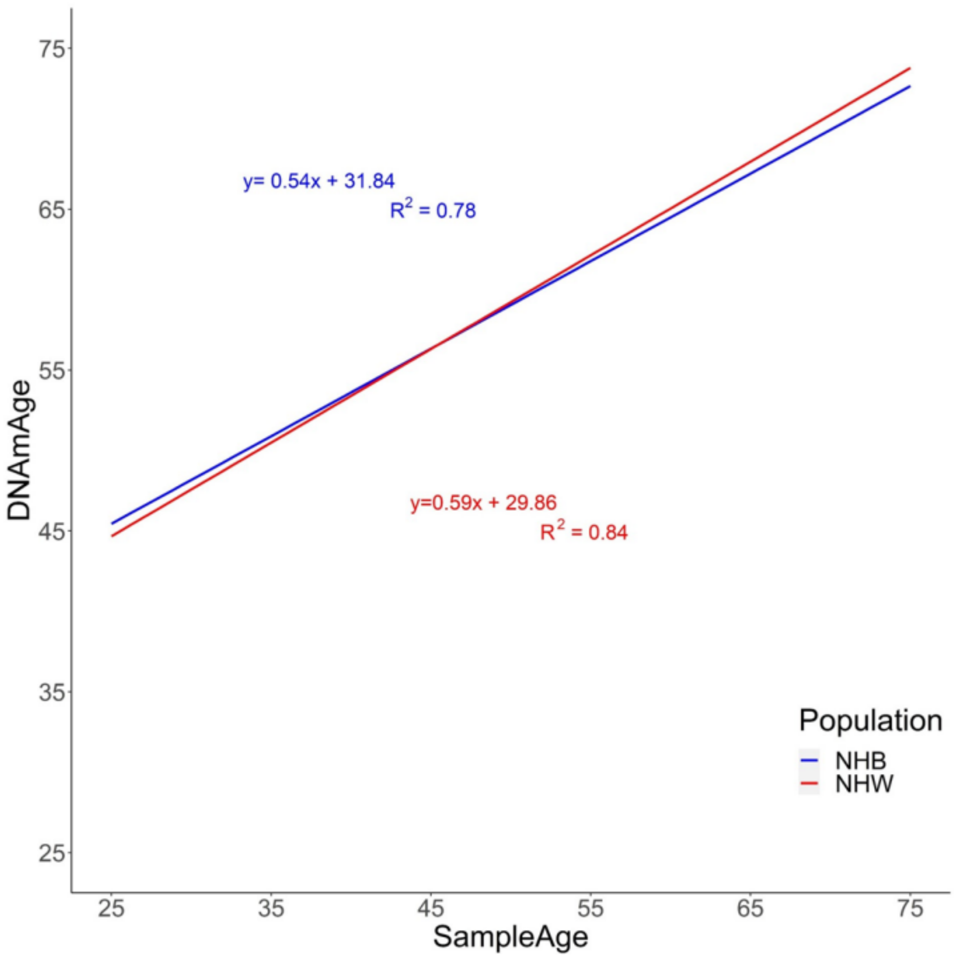

eFigure 2. Linear Correlation Between Epigenetic Age Based on Horvath's Clock and Chronological Age Among NHB and NHW Survivors Respectively

© 2024 Chen C et al. *JAMA Network Open*

eTable 5. General Linear Model of EAA_Horvath Among All Survivors

**Coefficients**	**Estimate**	**95% CI**	**P**
**Intercept**	-0.68	-1.05, -0.31	**<0.001**
**NHB vs. NHW**	0.44	0.01, 0.87	**0.05**
**Male vs. female**	0.36	0.07, 0.65	**0.02**
**Chest radiotherapy**	1.19	0.88, 1.50	**< 0.001**
**Alkylating agents**	0.44	0.15, 0.73	**0.004**
**Epipodophyllotoxins**	0.00	-0.33, 0.33	0.99
**BMI (>=25.0 vs. <25.0)**	0.01	-0.30, 0.32	0.95
**Smoking status (former/current vs. never)**	-0.47	-0.76, -0.18	**0.002**

Abbreviations: EAA, epigenetic age acceleration; NHB, non-Hispanic Black; NHW, non-Hispanic White.

© 2024 Chen C et al. *JAMA Network Open*

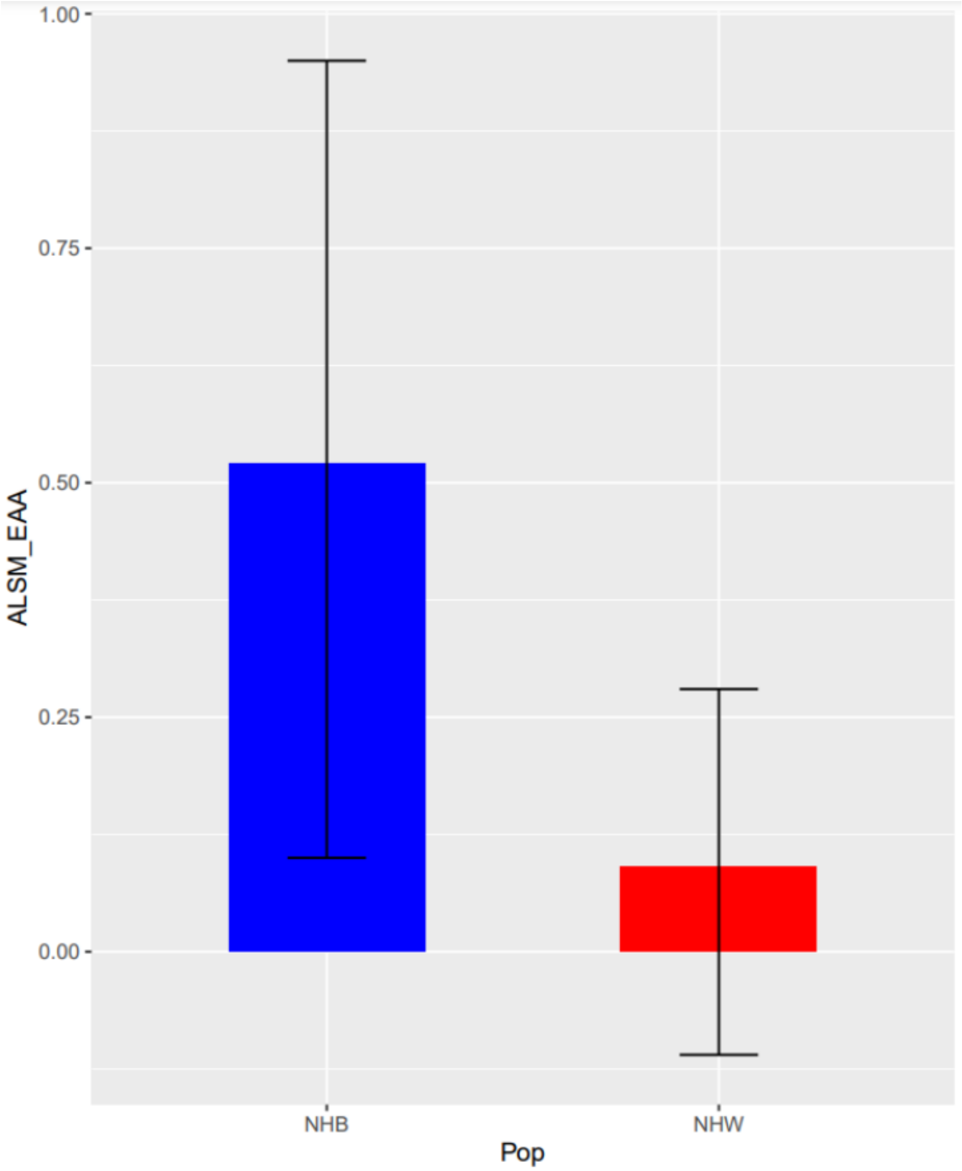

eFigure 3. Adjusted Least Square Mean (ALSM) of Epigenetic Age Acceleration (EAA) Based on Horvath's Clock Among NHB and NHW Survivors

General linear regression models were analyzed for the association of EAA-Horvath with racial groups (NHB vs. NHW) while adjusting for sex, chest-RT, alkylating agents, epipodophyllotoxins, BMI and smoking.

© 2024 Chen C et al. *JAMA Network Open*

eTable 6. Stratified Analysis of EAA-Horvath by Racial/Ethnic Groups for Treatment Exposures

**Racial/ethnic group**	**NHW**			**NHB**		
**Coefficients**	Estimate	95% CI	P	Estimate	95% CI	P
**Intercept**	-0.54	-0.93, -0.15	0.007	-1.43	-2.37, -0.49	0.02
**Male vs. female**	0.31	0.00, 0.62	0.05	1.08	0.14, 2.02	0.03
**Chest radiotherapy**	1.21	0.88, 1.54	<0.001	0.94	-0.08, 1.96	0.07
**Alkylating agents**	0.40	0.09, 0.71	0.01	0.70	-0.24, 1.64	0.10
**Epipodophyllotoxins**	-0.01	-0.36, 0.34	0.96	0.18	-0.90, 1.26	0.70
**BMI (>=25.0 vs. <25.0)**	-0.12	-0.43, 0.19	0.47	1.09	0.09, 2.09	0.03
**Smoking status (former/current vs. never)**	-0.47	-0.78, -0.16	0.003	-0.42	-1.46, 0.62	0.4

Abbreviations: EAA, epigenetic age acceleration; NHB, non-Hispanic Black; NHW, non-Hispanic White.

© 2024 Chen C et al. *JAMA Network Open*

eTable 5. rs732314 is Associated With EAA-Horvath Among All Survivors

Coefficients	Estimate	SE	P
**Intercept**	-1.39	-1.74, -1.04	**<0.001**
**NHB vs. NHW**	0.35	-0.08, 0.78	0.12
**Male vs. female**	0.36	0.07, 0.65	**0.010**
**rs732314**	0.42	0.22, 0.62	**<0.001**
**Chest radiotherapy**	1.29	0.98, 1.60	**<0.001**
**Alkylating agents**	0.50	0.21, 0.79	**<0.001**

Abbreviations: EAA, epigenetic age acceleration; NHB, non-Hispanic Black; NHW, non-Hispanic White.

© 2024 Chen C et al. *JAMA Network Open*
