# Supplementary material for: Race and Ethnicity, Socioeconomic Factors, and Epigenetic Age Acceleration in Survivors of Childhood Cancer
Source: JAMA Netw Open. 2024 Jul 2;7(7):e2419771. doi: 10.1001/jamanetworkopen.2024.19771 (PMC11220564; doi:10.1001/jamanetworkopen.2024.19771)
Supplement: Supplement 2. — Data Sharing Statement [file jamanetwopen-e2419771-s002.pdf]

Data Sharing Statement

Chen. Raceand Ethnicity, Socioeconomic Factors, and Epigenetic Age Acceleration in Survivors of Childhood Cancer. *JAMA Netw Open*. Published July 02, 2024. doi:10.1001/jamanetworkopen.2024.19771

Data
Data available: Yes
Data types: Deidentified participant data
How to access data: https://stjude.cloud
When available: With publication

Supporting Documents
Document types: None

Additional Information
Who can access the data: researchers whose proposed use of the data has been approved
Types of analyses: for a specified purpose
Mechanisms of data availability: after approval of a proposal
